# Investigation of the role of *AcTPR2* in kiwifruit and its response to *Botrytis cinerea* infection

**DOI:** 10.1186/s12870-020-02773-x

**Published:** 2020-12-10

**Authors:** Zhe-Xin Li, Jian-Bin Lan, Yi-Qing Liu, Li-Wang Qi, Jian-Min Tang

**Affiliations:** 1 Chongqing Key Laboratory of Economic Plant Biotechnology, Collaborative Innovation Center of Special Plant Industry in Chongqing, College of Landscape Architecture and Life Science/ Institute of Special Plants, Chongqing University of Arts and Sciences, Yongchuan, 402160 P.R. China; 2 State Key Laboratory of Tree Genetics and Breeding, Research Institute of Forestry, Chinese Academy of Forestry, Beijing, 100091 P.R. China

**Keywords:** *AcTPR2*, *Botrytis cinerea*, IAA signaling, Kiwifruit, Virus-induced gene silencing

## Abstract

**Background:**

Elucidation of the regulatory mechanism of kiwifruit response to gray mold disease caused by *Botrytis cinerea* can provide the basis for its molecular breeding to impart resistance against this disease. In this study, ‘Hongyang’ kiwifruit served as the experimental material; the TOPLESS/TOPLESS-RELATED (TPL/TPR) co-repressor gene *AcTPR2* was cloned into a pTRV2 vector (*AcTPR2*-TRV) and the virus-induced gene silencing technique was used to establish the functions of the *AcTPR2* gene in kiwifruit resistance to *Botrytis cinerea*.

**Results:**

Virus-induced silencing of *AcTPR2* enhanced the susceptibility of kiwifruit to *Botrytis cinerea*. Defensive enzymes such as superoxide dismutase (SOD), peroxidase (POD), catalase (CAT), and phenylalanine ammonia-lyase (PAL) and endogenous phytohormones such as indole acetic acid (IAA), gibberellin (GA_3_), abscisic acid (ABA), and salicylic acid (SA) were detected. Kiwifruit activated these enzymes and endogenous phytohormones in response to pathogen-induced stress and injury. The expression levels of the IAA signaling genes—*AcNIT*, *AcARF1*, and *AcARF2*—were higher in the *AcTPR2*-TRV treatment group than in the control. The IAA levels were higher and the rot phenotype was more severe in *AcTPR2*-TRV kiwifruits than that in the control. These results suggested that *AcTPR2* downregulation promotes expression of IAA and IAA signaling genes and accelerates postharvest kiwifruit senescence. Further, *Botrytis cinerea* dramatically upregulated *AcTPR2,* indicating that *AcTPR2* augments kiwifruit defense against pathogens by downregulating the IAA and IAA signaling genes.

**Conclusions:**

The results of the present study could help clarify the regulatory mechanisms of disease resistance in kiwifruit and furnish genetic resources for molecular breeding of kiwifruit disease resistance.

**Supplementary Information:**

The online version contains supplementary material available at 10.1186/s12870-020-02773-x.

## Background

Kiwifruit (*Actinidia chinensis* L.) is prone to fungal pathogen infections that cause major postharvest crop losses and may render the fruit unsafe for consumers. *Botrytis cinerea* (*B. cinerea*) is a fungal pathogen responsible for gray mold. It can damage or destroy ≤30% of the kiwifruit crop [1]. In order to breed gray mold resistance into kiwifruit, it is first necessary to elucidate the mechanism regulating plant pathogen response. Current research on gray mold control has focused mainly on physical, chemical, and certain biological controls [2–5]. However, the regulatory and signaling pathways associated with the genes controlling disease resistance in kiwifruit remain to be determined.

Members of the TOPLESS/TOPLESS-RELATED (TPL/TPR) co-repressor protein family interact with transcription factors [6]. The TPL/TPR domains include the highly conserved N-terminal TPD region comprising the lissencephaly homologous (LisH) dimerization motif, a C-terminal to the LisH (CTLH) motif, and C-terminal WD40-repeats [7, 8]. The TOPLESS domains (TPDs) mediate TPL/TPR oligomerization and interact with proteins containing the EAR motif [9]. Most EAR motifs were detected in proteins regulating the transcription of signaling pathways for phytohormones such as auxin, abscisic acid, gibberellins, salicylic acid, ethylene, and jasmonate [10–14].

It was first reported that TPL/TPR co-repressors directly interact with the WUSCHEL (WUS) transcription factor in *Arabidopsis thaliana* [6]. The TPL/TPR co-repressor family members, TPL and TPR4, interact with WUS. Five TPL/TPR family genes including *TPL* and *TPR1–TPR4* were detected in *Arabidopsis* [15]. The TPL/TPR co-repressors play vital roles in plant growth and development [10, 16–18]. A defective *tpl* mutation, nonetheless, permits normal embryonic development in *Arabidopsis*. Hence, TPL protein function is redundant and may be replaced by four other homologous proteins. When *tpl*/*tpr1/tpr3*/*tpr4* quadruple mutant lines were transformed with TPR2 protein RNAi, abnormal embryonic development occurred. Thus, *tpl* is a dominant negative mutation for multiple *TPL*-related proteins [15].

Overexpression of the IAA metabolism-related genes *OsGH3.1* and *OsCYP71Z2* significantly improved rice resistance to bacterial blight caused by *Xanthomonas oryzae* pv. *oryzae*. Therefore, the IAA signaling pathway is implicated in plant defense [19, 20]. TPL/TPR interact with the transcription complexes involved in auxin signal transduction. Auxins induce the formation of ternary repressor-phytohormone-E3 ligase complexes, which, in turn, cause E3 ligase-catalyzed repressor protein ubiquitination and degradation, and upregulate phytohormone target genes [7]. Auxin-mediated TIR1 (transport inhibitor response 1) E3 ubiquitin ligase binding causes ubiquitination and proteolysis of Aux/IAA repressor protein. At low auxin levels, IAA repressors recruit TPL/TPR to Auxin response factor (ARF) in order to prevent the expression of ARF and its target genes [21, 22].

A proteomic analysis showed that *AcTPR2* was highly upregulated in kiwifruits following *B. cinerea* infection [23]. Here, we used the ‘Hongyang’ kiwifruit cultivar as the experimental material. We cloned *AcTPR2* and used the virus-induced gene silencing (VIGS) technique to establish the roles of *TPR2* in kiwifruit resistance to *B. cinerea*. We also ran an expression analysis to investigate possible interactions among *AcTPR2* and the IAA signaling genes. The results of the present study could help clarify the resistance mechanism responsible for disease resistance in kiwifruit, and furnish genetic resources for molecular breeding of kiwifruit disease resistance.

## Results

### Construction of the *AcTPR2*-TRV2 vector

**Fig. 1:**
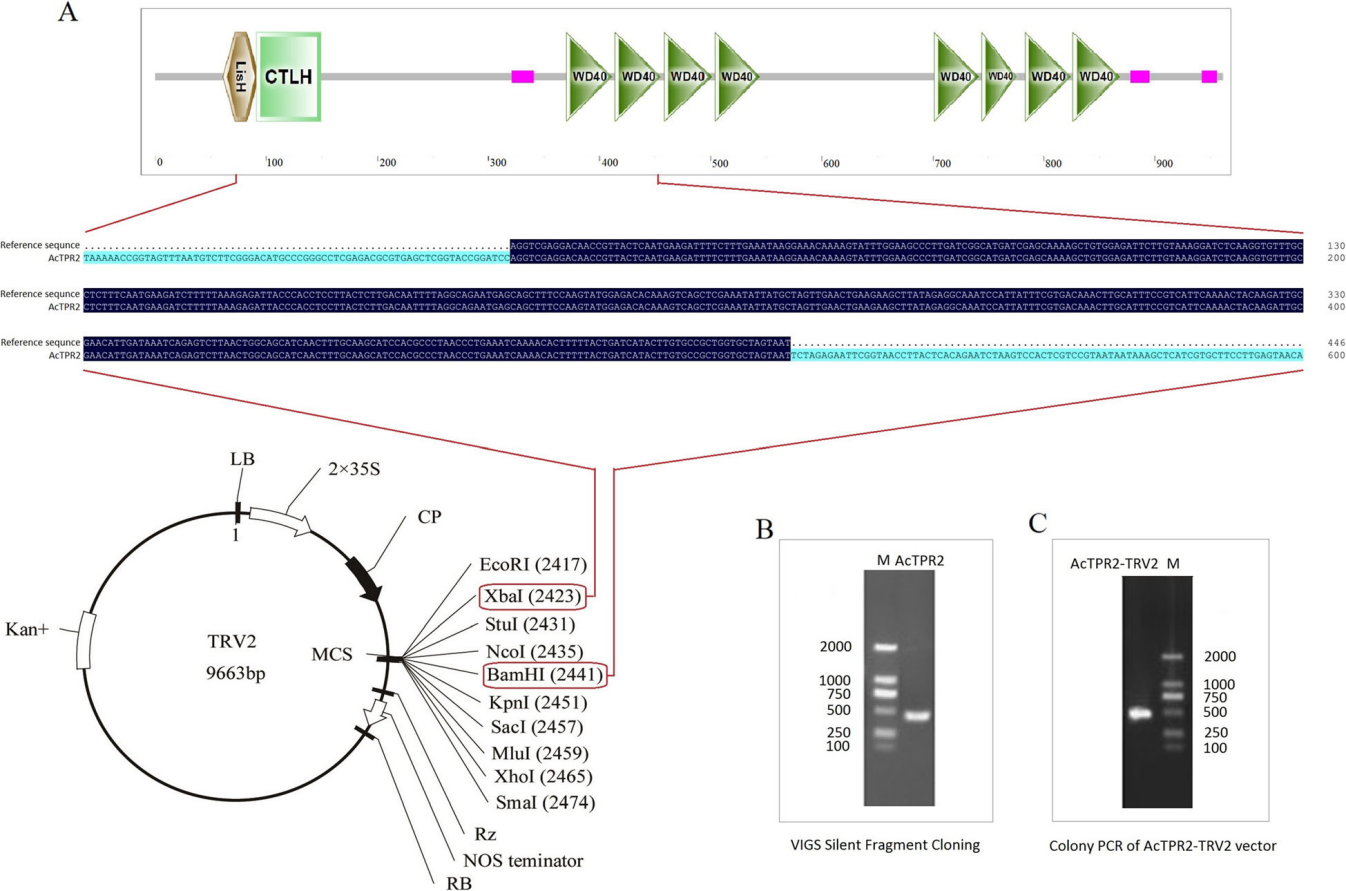
Construction of *AcTPR2*-TRV2. **a** Sructure analysis of the cloned *AcTPR2* in kiwifruits, and the target gene fragment constructed to the pTRV2 vector. **b** PCR results of the target gene fragment. **c** PCR results of *AcTPR2*-TRV2 vector

A silencing fragment was cloned and its sequence was the same as that of the reference *AcTPR2* (Ach25g228601.2-TA, http://kiwifruitgenome.org/). An *AcTPR2*-TRV2 construct was generated by introducing a 446-bp *Xba*I/*Bam*HI DNA fragment into a pTRV2 vector (Fig. 1a and b). PCR detection of resistant colonies bearing the *AcTPR2*-TRV2 construct was performed to confirm that the silencing fragment was successfully ligated onto the pTRV2 vector (Fig. 1c).

### *AcTPR2* expression was greatly reduced in *AcTPR2*-TRV fruits

**Fig. 2:**
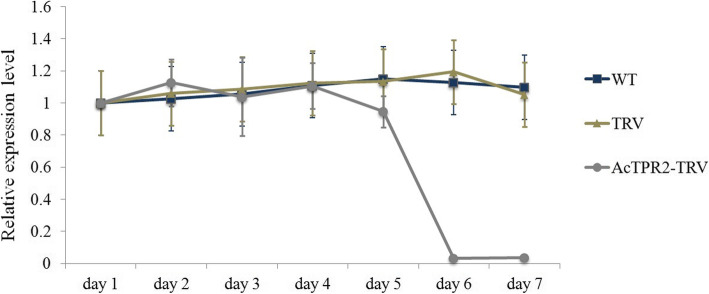
Relative expression level of *AcTPR2* in fruits transformed with ddH_2_O water, empty vector and *AcTPR2*-TRV at 1–7 days. Values are means±SE of three biological replicates

Agrobacterium GV3101 harboring the *AcTPR2*-TRV2 expression vector (*AcTPR2*-TRV), sterilized ddH_2_O (WT), and the vector pTRV1–2 (TRV), were transformed to ‘Hongyang’ kiwifruit by transient injection. *AcTPR2* expression was measured for all three groups. *AcTPR2* level was markedly downregulated in the *AcTPR2*-TRV fruits at 6 days post-injection. In contrast, there were no drastic differences in *AcTPR2* expression between the WT and TRV groups (Fig. 2).

### Virus-induced silencing of *AcTPR2* enhanced kiwifruit susceptibility to *B. cinerea*

**Fig. 3:**
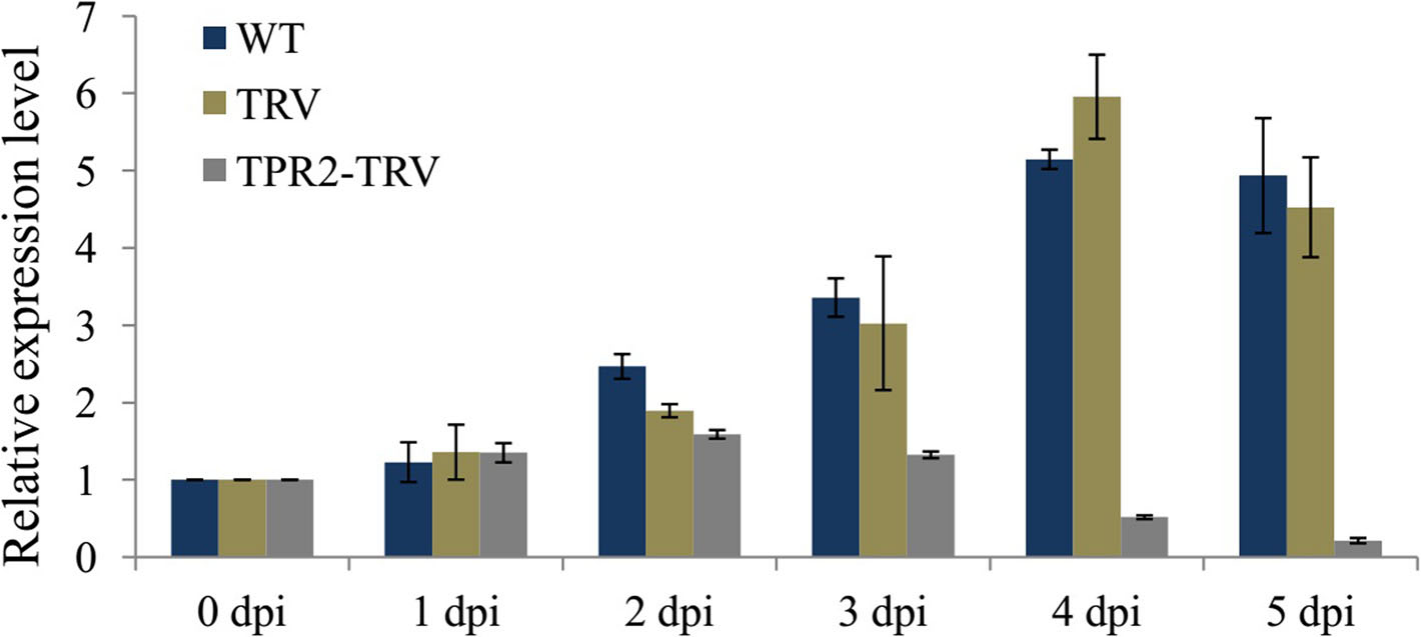
Relative expression level of *AcTPR2* in fruits transformed with ddH_2_O water, empty vector and *AcTPR2*-TRV upon infection of *B. cinerea*. Values are means±SE of three biological replicates

*AcTPR2* expression was compared between *AcTPR2*-TRV and control kiwifruit after *B. cinerea* infection*.* The *AcTPR2* level was highest at 4 days post-inoculation (dpi) and the infection time was prolonged in the WT, TRV empty vector, and *AcTPR2*-TRV fruits. The *AcTPR2* levels in these treatments were nearly sixfold higher than they were in the control at 1 dpi. However, the *AcTPR2* level in *AcTPR2*-TRV was twofold lower than it was at 1 dpi (Fig. 3).

**Fig. 4:**
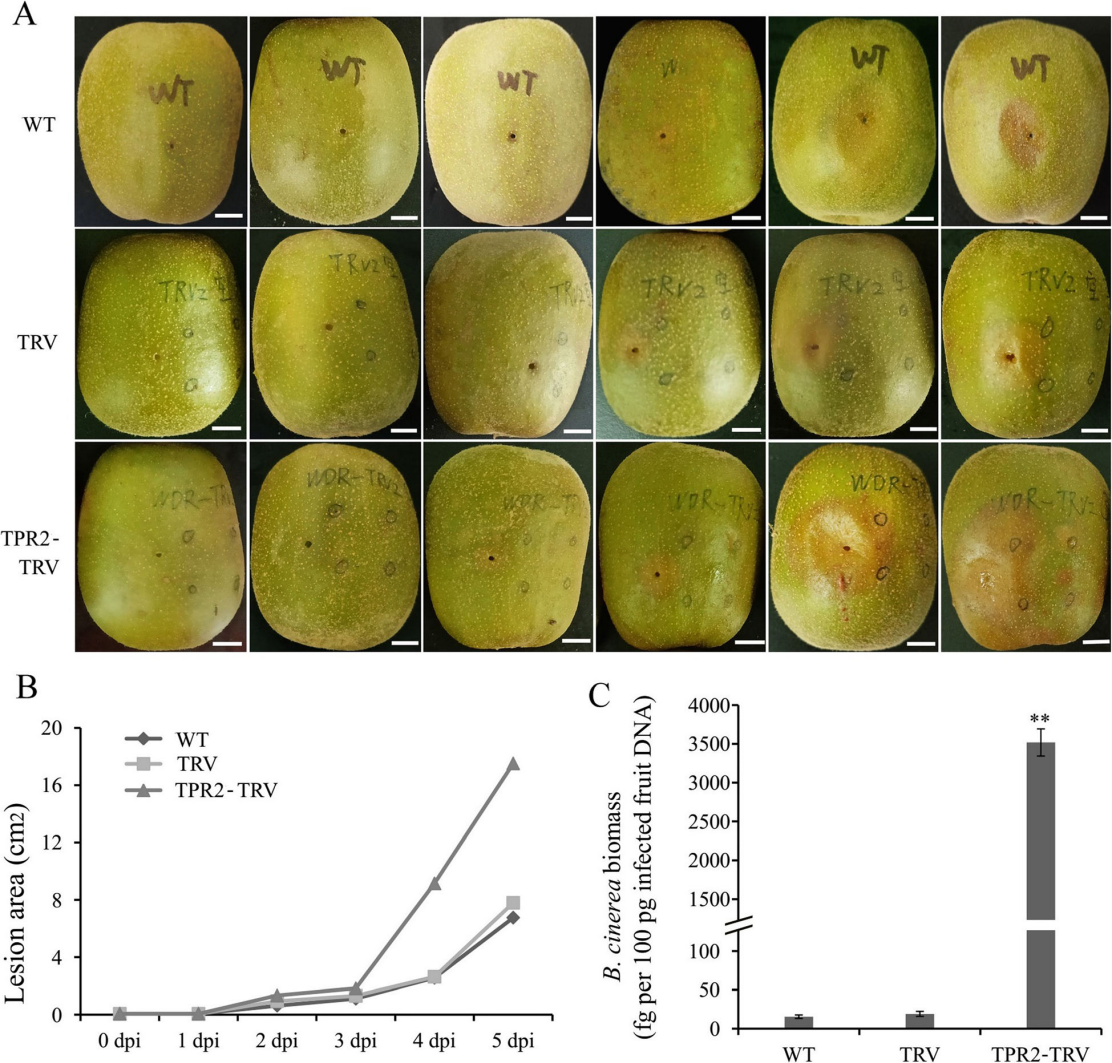
**a** Appearance and quality of kiwifruit upon *B. cinerea* infection. **b** Lesion area of kiwifruits of WT, TRV and *AcTPR2*-TRV. Values are means±SE of 10 biological replicates

The lesion areas on *AcTPR2*-silenced kiwifruit were larger than those for the control groups. Furthermore, the injection sites were more susceptible to rot in the *AcTPR2*-silenced kiwifruit than they were in the control. At 5 dpi, the lesion areas on the *AcTPR2*-TRV fruit were nearly threefold larger than they were on the WT and TRV vector fruits*.* The *B. cinerea* load was also significantly higher in the *AcTPR2*-TRV fruits than that in the control at 4 dpi and 5 dpi. Thus, virus-induced silencing of *AcTPR2* enhanced kiwifruit susceptibility to *B. cinerea* (Fig. 4).

### Kiwifruit activate pathogen resistance-related defense enzymes in response to infection

**Fig. 5:**
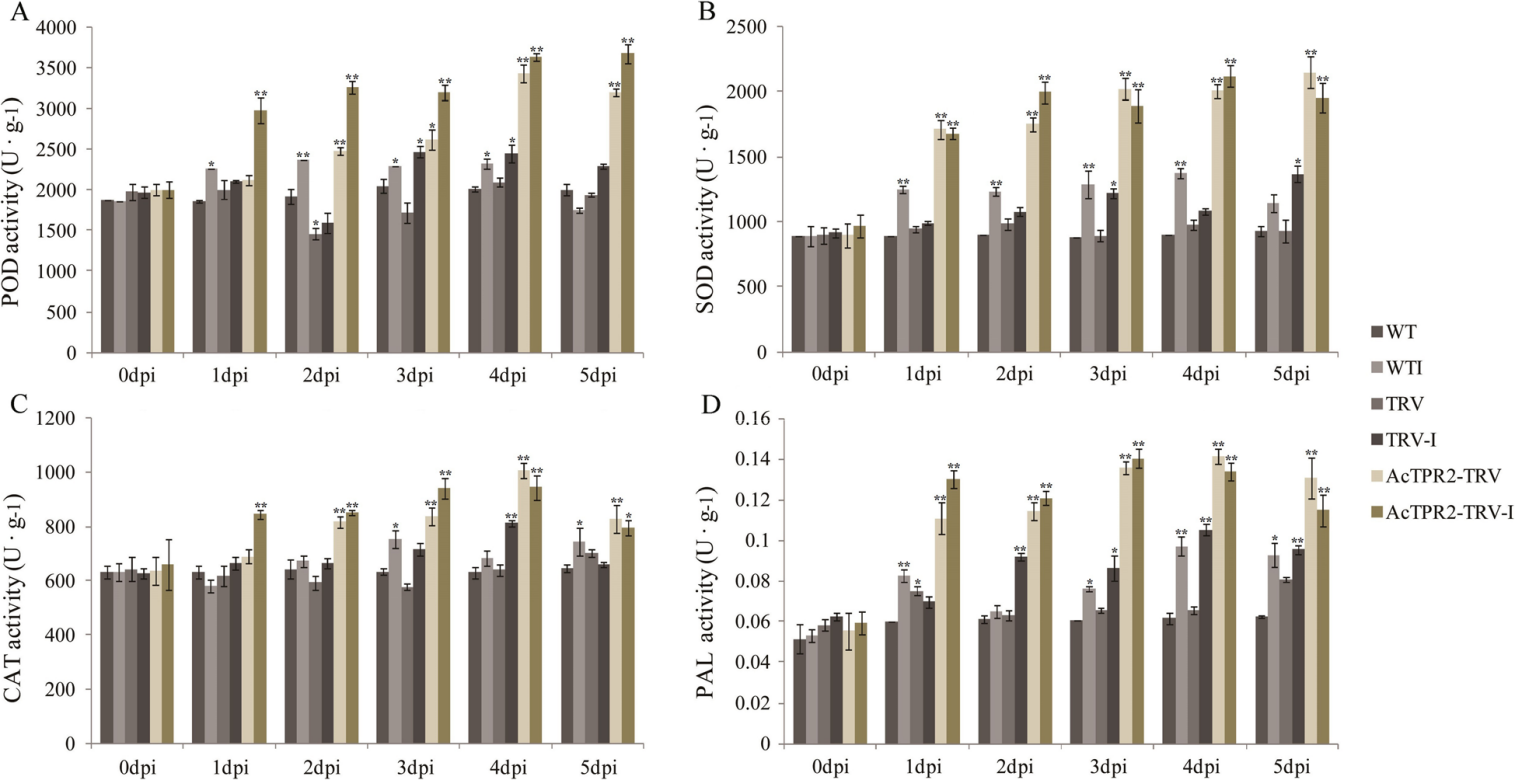
Effects of *B. cinerea* infection on activities of 4 defense enzymes in kiwifruit of WT, TRV and *AcTPR2*-TRV. The activity of antioxidant enzymes including SOD, POD, CAT and PAL were detected. Values are means±SE of three biological replicates. “*” represent the significant difference (*p* < 0.05), while “**” represent the extremely significant difference (*p* < 0.01)

The activity levels of enzymes such as SOD, POD, CAT, and PAL are indicative of plant disease resistance. These enzymes may be upregulated in response to biotic and abiotic stress and enhance host resistance. The activity levels of all four defense enzymes increased in the control and *AcTPR2*-TRV kiwifruits in response to *B. cinerea* infection. SOD, POD, and PAL rapidly reacted to pathogenesis at 1 dpi and their activity levels continued to rise until 4 dpi, but decreased by 5 dpi. CAT was first induced at 2 dpi and its activity steadily increased with infection time. However, at 5 dpi, its activity declined. In the absence of *B. cinerea* infection, the activity levels of all four enzymes were much higher in *AcTPR2*-TRV than they were in the control. Nevertheless, the enzyme activity markedly increased in the *AcTPR2*-TRV groups under infection stress (Fig. 5).

### Increased *B. cinerea* susceptibility in the *AcTPR2*-TRV groups is related to phytohormone interactions

**Fig. 6:**
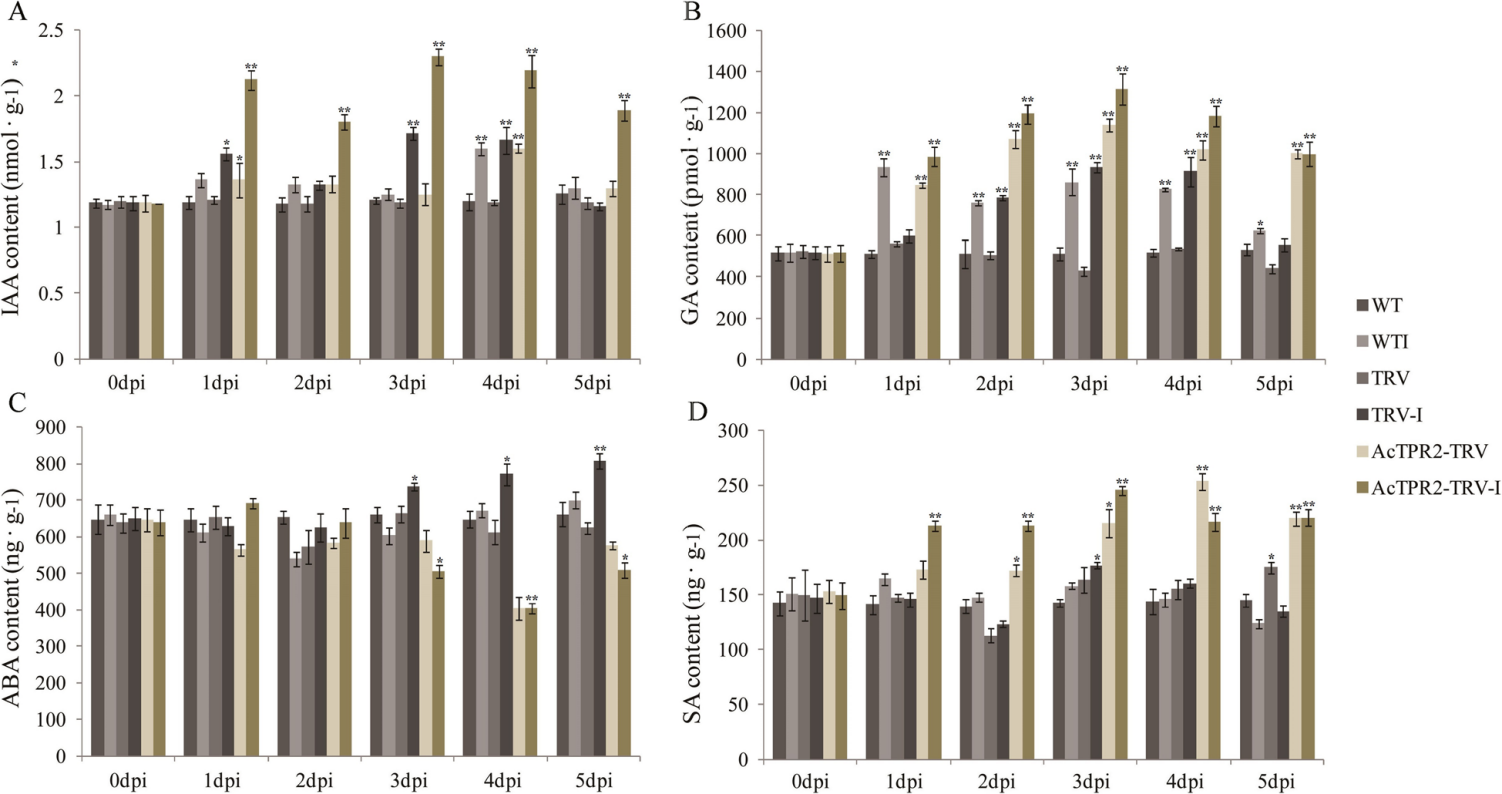
Effects of *B. cinerea* infection on contents of 4 endogenous hormones in kiwifruit of WT, TRV and *AcTPR2*-TRV. The content of phytohormones including of IAA, ABA, GA_3_ and SA were detected. Values are means±SE of three biological replicates. “*” represent the significant difference (*p* < 0.05), while “**” represent the extremely significant difference (*p* < 0.01)

TPL/TPR proteins participate in plant signaling pathways and the various phytohormones interact. Here, the relative levels of the phytohormones, IAA, GA_3_, ABA, and SA, were measured. IAA, GA, and SA levels were observed to sharply increase before 4 dpi, and thereafter, they decreased rapidly. In the absence of *B. cinerea* infection, IAA, GA, and SA levels were substantially higher in *AcTPR2*-TRV than those in the control WT and TRV groups. Upon infection, the levels of IAA, GA, and SA were higher in *AcTPR2*-TRV than they were in the uninfected *AcTPR2*-TRV at 1–4 dpi. However, the levels of these phytohormones declined at 5 dpi (Fig. 6a, b, and d). In the control, the ABA content continuously increased as the *Botrytis cinerea* infection prolonged. In case of the *AcTPR2*-TRV treatment at 1–2 dpi, the ABA content was higher following *B. cinerea* infection than that in the uninfected fruits. After 2 dpi, however, the ABA level considerably fell in the *AcTPR2*-TRV kiwifruit (Fig. 6c).

### Virus-induced silencing of *AcTPR2*-induced IAA signaling gene expression

**Fig. 7:**
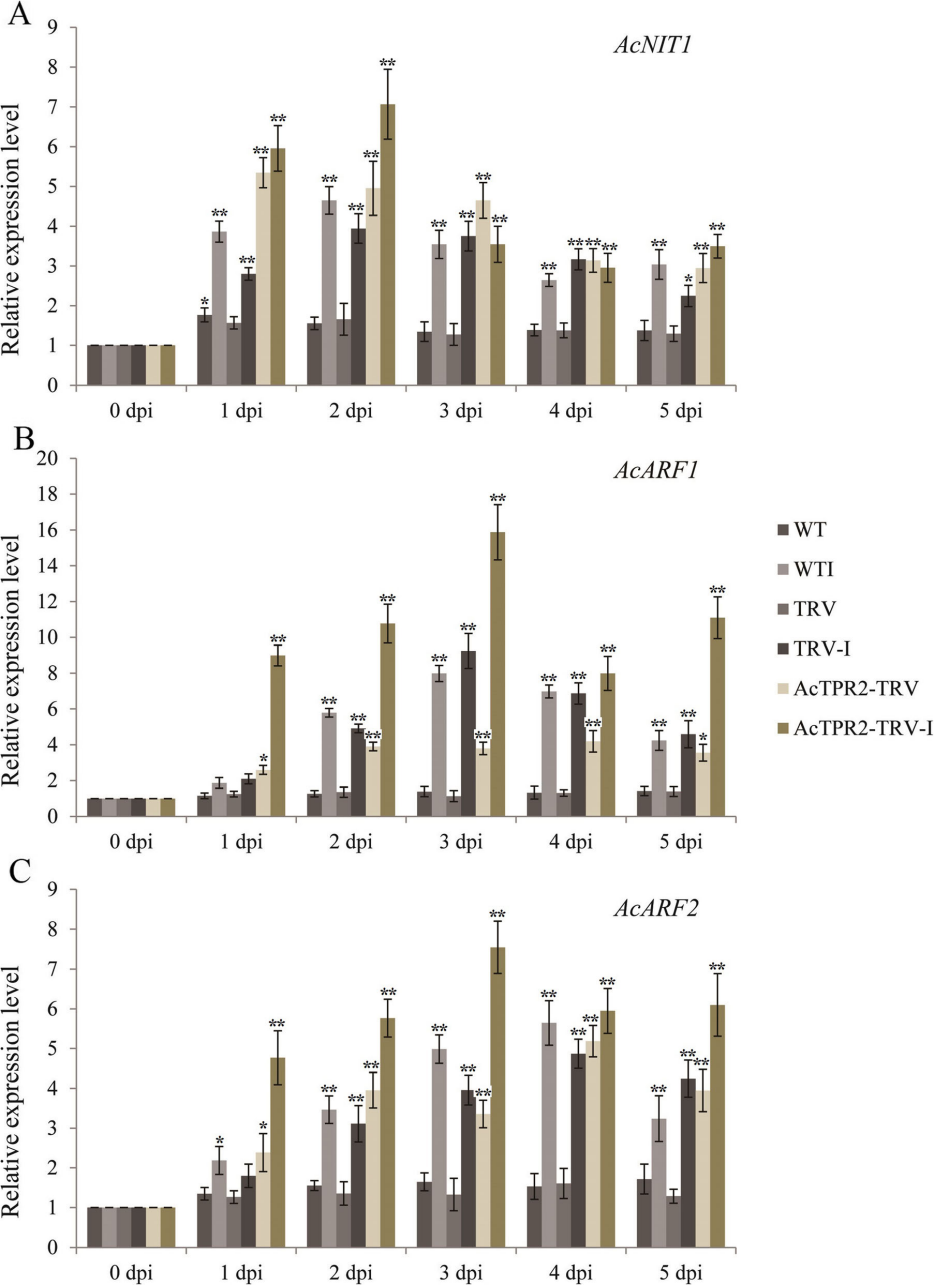
Expression analysis of IAA signaling genes in kiwifruits of WT, TRV and *AcTPR2*-TRV upon *B. cinerea* infection. Values are means±SE of three biological replicates. “*” represent the significant difference (*p* < 0.05), while “**” represent the extremely significant difference (*p* < 0.01)

Here, we used qRT-PCR to measure the expression levels of the genes governing auxin biosynthesis and signaling in the *AcTPR2*-TRV and control fruits. *AcNIT1* was slightly upregulated in the control fruit within 5 days of storage, but markedly increased upon *B. cinerea* infection, especially at 1–3 dpi. The *AcNIT1* level was higher in the *AcTPR2*-TRV than that in the control fruits (Fig. 7a). *AcARF1*and *AcARF2* were somewhat upregulated in the control but their levels were fourfold higher in the *AcTPR2* silenced fruits than those in the control. *AcARF1*and *AcARF2* were strongly induced in response to *B. cinerea* infection (Fig. 7b and c).

## Discussion

TPL/TPR expression may be associated with plant-pathogen interactions [24]. To the best of our knowledge, however, no prior study has explored the roles of TPL/TPR in kiwifruit-*B. cinerea* interaction. Previous research showed that *AcTPR2* is highly expressed in kiwifruit following *B. cinerea* infection [23], and therefore, it can potentially play a crucial role in this process. Here, we applied expression profiling, transgenic studies, and infection analysis to investigate the functions of *AcTPR2* in disease resistance in kiwifruit-*B. cinerea* interactions.

We used the VIGS method to examine the reverse function of *AcTPR2* in kiwifruit resistance to *B. cinerea*. A qRT-PCR analysis showed that *AcTPR2* was downregulated in *AcTPR2*-TRV2 kiwifruits 6 days after transformation and the transformed vectors had a silencing effect (Fig. 2). *AcTPR2* was markedly upregulated in the kiwifruits after *B. cinerea* infection. Hence, *AcTPR2* may have a defensive function against *B. cinerea* in kiwifruit (Fig. 3). However, *AcTPR2* expression was substantially lower in the *AcTPR2*-TRV2 kiwifruits than that in the controls. The foregoing results validated the silencing effect identified by VIGS.

*TPR2* overexpression in plants might enhance pathogen resistance. Nevertheless, little research has been conducted on the effects of *TPR2* downregulation [24]. Here, we provided reverse evidence for the role for *TPR2* in fungal pathogen resistance in kiwifruit. Virus-induced silencing of *AcTPR2* increased *B. cinerea* susceptibility in kiwifruit. These findings were consistent with a previous study reporting that *AtTPR1* overexpression activated *Arabidopsis* defense responses, even though various *TPRs* were redundant [24].

Activation of a defense response is accompanied by the induction of pathogenesis-related enzymes. Stress induces excessive generation of reactive oxygen species (ROS). SOD, POD, and CAT are vital plant antioxidant enzymes that remove active oxygen produced in response to external damage [25]. PAL is a critical enzyme in the phenylpropane metabolic pathway, which generates various secondary metabolites and prevents pathogen invasion [26]. Here, all four defense enzymes were upregulated in the control and the *AcTPR2*-TRV2 kiwifruits in the presence of *Botrytis cinerea*. The activity levels of SOD, POD, CAT, and PAL increased in response to the pathogen infection until 4 dpi and decreased thereafter. The onset of infection at 4 dpi might have indicated a breakdown in localized plant defense responses (Fig. 5). In the absence of infection, all four enzymes were markedly upregulated in *AcTPR2*-TRV2 as compared to those in the control. Under *B. cinerea* infection stress, however, the activity levels of all four enzymes drastically increased in the *AcTPR2*-TRV2 treatment (Fig. 5). Thus, after the *AcTPR2* gene was silenced, the kiwifruit internally upregulated these enzymes. Nevertheless, the *AcTPR2*-TRV2 kiwifruits were relatively more susceptible to rot.

TPL/TPR interact with the transcription complexes involved in the phytohormone pathways, especially in auxin signal transduction [7, 14]. Various phytohormones coordinate plant growth and stress adaptation. Here, in the absence of *Botrytis cinerea* infection, the IAA, GA, and SA levels were considerably higher in *AcPGIP*-TRV2 than those in the control. Therefore, *AcPGIP* silencing enables kiwifruit to activate phytohormone signaling pathways promptly via other mechanisms and defend itself against *B. cinerea*. This finding was consistent with that of a previous study stating that plants produce endogenous phytohormones that adjust the pathogen response [20]. The levels of all endogenous phytohormones in *AcPGIP*-TRV2 increased but then decreased to lower levels than those of the control. Further, there were synergies among IAA, GA, and SA. The ABA content was higher in the fruit at 1–2 dpi than that in the uninfected fruit. However, the ABA level dramatically decreased after 2 dpi. This pattern was not observed for the other three phytohormones.

**Fig. 8:**
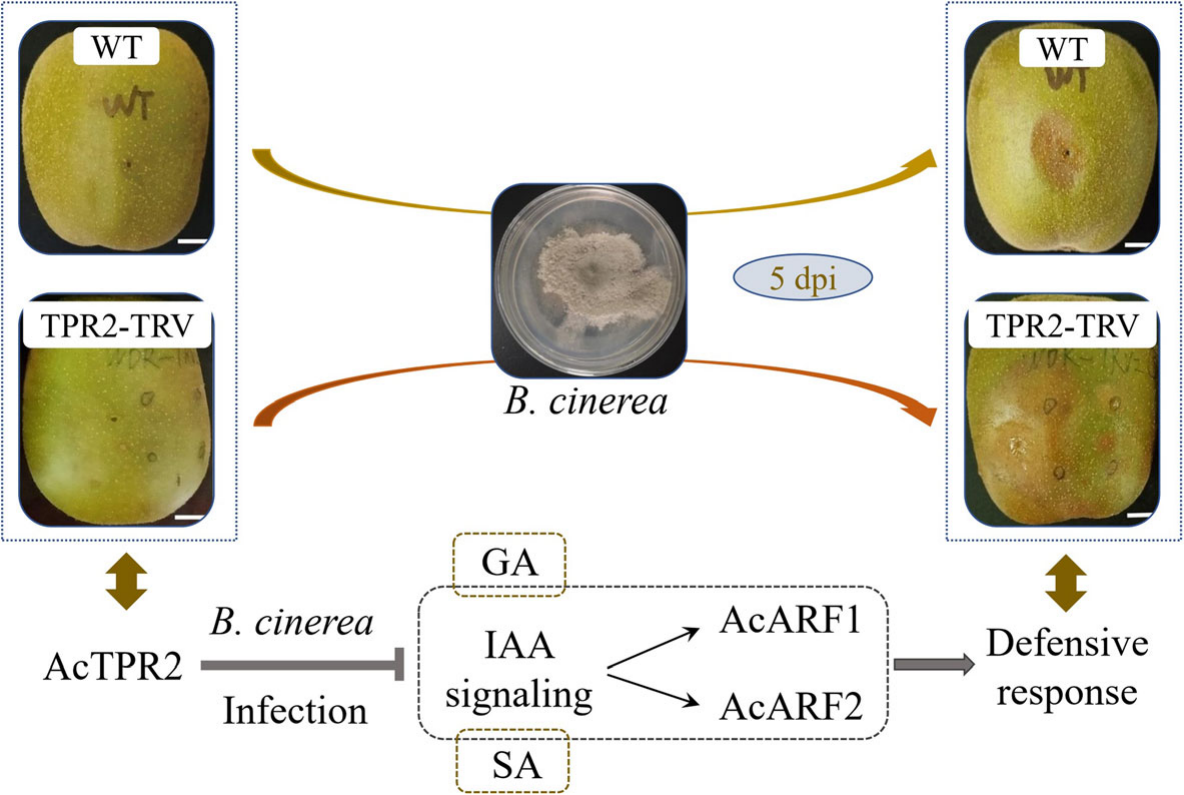
A proposed model for the present study. *AcTPR2* could enhance the defensive ability of kiwifruits against *B. cinerea* by negatively regulate IAA and IAA signaling genes. While GA and SA have synergistic effect with IAA, and jointly respond to pathogen infection

In this study, we evaluated the expression levels of three IAA signaling genes. Prolonged storage slightly increased *AcNIT1* in the control fruit. Thus, IAA participated in fruit ripening and senescence. *Botrytis cinerea* infection accelerated fruit senescence by inducing host IAA biosynthesis and promoting expression of IAA signaling genes such as *AcNIT1*, *AcARF1*, and *AcARF2.* Moreover, the expression levels of all three genes were higher in AcTPR2-TRV than those in the control. There were relatively elevated IAA levels and a more severe rot phenotype in the *AcTPR2*-TRV kiwifruit. Hence, *AcTPR2* downregulation might promote the expression of IAA and IAA signaling genes and accelerate postharvest kiwifruit senescence. Further, *Botrytis cinerea* infection drastically increased the relative *AcTPR2* responses. We, therefore, propose that *AcTPR2* enhances plant pathogen defense by downregulating IAA and IAA signaling genes (Fig. 8). The results of the present study are consistent with those of a previous research reporting that *Arabidopsis* overexpressing *AtTRP1* showed a comparatively weak response to exogenous IAA and demonstrated altered expression of a subset of auxin early response genes [27].

## Conclusion

The virus-induced gene silencing technique was used to establish the functions of the *AcTPR2* gene in kiwifruit resistance to *Botrytis cinerea*. Virus-induced silencing of *AcTPR2* increased kiwifruit sensitivity to *B. cinereal* infestations. The antioxidant enzymes SOD, POD, CAT, and PAL and the endogenous phytohormones IAA, GA, ABA, and SA were all activated in response to pathogen-induced stress and injury in kiwifruit. The IAA signaling genes *AcNIT*, *AcARF1*, and *AcARF2* were all upregulated in kiwifruit transformed by AcTPR2-TRV compared to the control. Since IAA levels were also higher and the gray mold rot phenotype was more intense in *AcTPR2*-TRV kiwifruits than they were in the control, *AcTPR2* downregulation promotes the IAA and IAA signaling genes and accelerates senescence in postharvest kiwifruit. *Botrytis cinerea* substantially upregulated *AcTPR2* in the kiwifruit compared with the control*.* Thus, *AcTPR2* augments kiwifruit defense against pathogens by downregulating the IAA and IAA signaling genes.

## Methods

### Plant materials

Kiwifruit (*Actinidia chinensis* cv. Hongyang) is a fruit crop of high economic importance and has interested consumers [28]. The variety passed the approval of the Sichuan Provincial Crop Variety Approval Committee and was named Kiwifruit ‘Hongyang’ in 1977. Jianmin Tang undertook the formal identification of the plant material used in the present study [3]. Nearly mature, undamaged, and pest-free kiwifruit were harvested at 130 days after flowering in an experimental facility in Kaizhou District, Chongqing, China (31°23′ N, 108°39′ E). Kiwifruit with an average weight of 95 g were transported to the laboratory within 5 h of harvest. Fruits were further disinfected with 2% (v/v) sodium hypochlorite for 2 min, rinsed with tap water, and air-dried.

### Vector constructions and transformation

Total RNA was extracted from the kiwifruit using RNAiso Plus reagents (*Takara* Biomedical Technology Co. Ltd., Beijing, China) and reverse-transcribed into cDNA with a PrimeScript™ RT Reagent Kit (*Takara* Biomedical Technology Co. Ltd., Beijing, China). The single electrophoretic band of the *AcTPR2* PCR product was recovered and cloned into a pMD®19-T Simple Vector (*Takara* Biomedical Technology Co. Ltd., Beijing, China). PCR-positive bacterial colonies was sequenced to verify *AcTPR2* correctness. An *AcTPR2*-TRV2 construct was generated by introducing a 446-bp *Xba*I/*Bam*HI DNA fragment into a pTRV2 vector (Fig. 1). The *AcTPR2* gene fragment and the pTRV2 vector were double-digested by *Xba*I/*Bam*HI, recovered, ligated, transformed into *E. coli* DH5α-competent cells, and incubated on a resistant plate medium containing 50 mg L^− 1^ kanamycin. Resistant colonies were detected by PCR and the PCR-positive bacterial colonies were selected to extract plasmids. The universal vector primer RV-XIAYOU (5-‘AACCTAAAACTTCAGACACG-3’) was used for sequencing. The *AcTPR2*-TRV2 sequence was the same as the reference sequence (Fig. 1). The *AcTPR2*-TRV2 expression vector was then introduced into *Agrobacterium tumefaciens* strain GV3101. Transformed *A. tumefaciens* harboring the pTRV2 vector was mixed in a 1:1 ratio with *A. tumefaciens* GV3101 bearing the pTRV1 vector. The mixed *Agrobacterium* cultures with density (OD600 = 1.0) were injected by syringe into kiwifruit. Sterilized ddH_2_O and vector TRV (pTRV1:pTRV2 = 1:1) served as the control.

### Infection by *B. cinerea*

Strain HFXC-16 of *B. cinerea* isolated from infected kiwifruit was incubated at 25 °C and grown on potato dextrose agar (PDA) for 2 weeks [29] (Chen et al., 2015). Seven days after injection of the fruit with *Agrobacterium tumefaciens*, sterilized ddH_2_O, or TRV1–2 vector, 10 μL *B. cinerea* spore suspension containing 10^4^ spores mL^− 1^ was injected by syringe into each wound induced near those formed by previous *Agrobacterium* or ddH_2_O injections. The treated kiwifruit were placed in covered plastic food trays, enclosed in polyethylene bags, and stored at 25 °C in a constant-temperature incubator (Sanyo Electric Co. Ltd., Osaka, Japan).

### *B. cinerea* DNA quantification

Genomic DNA was isolated from samples of *B. cinerea*-infected kiwifruit at 5 dpi. The genomic DNA of *B. cinerea* was tested using the universal primers ITS1/ITS4 and a 551-bp band was obtained. Fifty picograms DNA was subjected to qRT–PCR analysis of the ITS sequence using primers designed from the sequenced 551-bp fragment (Table 1).

**Table 1:** Primers used for PCR and qRT-PCR

Purpose	Name	Sequence (5′-3′)	
*AcTPR2*-pTRV2 construction	*AcTPR2*	F	**(XbaI)GCTCTAGA**ATTACTAGCACCAGCGGCAC
		R	**(BamHI)CGGGATCC**AGGTCGAGGACAACCGTTAC
PCR	*ITS*	F	TCCGTAGGTGAACCTGCGC
		R	TCCTCCGCTTATTGATATGC
qRT-PCR	*ITS*	F	CTGTTCGAGCGTCATTTCAA
		R	CCTACCTGATCCGAGGTCAA
qRT-PCR	*Actin*	F	GCAGTGTTTCCCAGTATTGT
		R	TCCATGTCATCCCAGTTGC
qRT-PCR	*AcTPR2*	F	GGGCTGCGTATACTGAGGAT
		R	TTGCTTGCTGTCAAAGGGTC
qRT-PCR	*AcNIT1*	F	TTTGTTTTGTCAGCCAACCA
		R	ATGCCCAACCACATCAAAAT
qRT-PCR	*AcARF1*	F	TTGTGTGCCATTAGGCATGT
		R	TGGTTTCCACTTGGTTAGGC
qRT-PCR	*AcARF2*	F	AGGGTCTTGCAAAGTCGAGA
		R	GATCGAGTCCCAAAACCTGA

### qRT-PCR for expression analysis

Kiwifruit tissues were collected daily for 6 days. The experimental design consisted of three replicates of 10 fruits per treatment and the experiment was repeated thrice. All tissues were stored at − 80 °C until RNA extraction. The total RNA was purified with RNAiso Plus Kits (TaKaRa Bio Inc., Shiga, Japan) following the manufacturer’s protocol. One microgram RNA was treated with RNase-free DNase I (Thermo Fisher Scientific, Waltham, MA, USA) to eliminate genomic DNA and reverse-transcribed with oligo (dT) using a TransScript All-in-One First-Strand cDNA Synthesis Supermix for qPCR (One-Step gDNA Removal) Kit (TransGen Biotech Co. Ltd., Beijing, China). A TB Green™ Premix Ex Taq™ (Tli RNaseH Plus; TaKaRa Bio Inc., Shiga, Japan) kit was used for qRT-PCR analysis. The *β-actin* gene served as an internal control [23]. Primers are listed in Table 1. The PCR products were cloned and sequenced to verify their identity.

### Analysis of defensive enzymes

One half gram kiwifruit tissue was extracted in 10 mL of 100 mM potassium phosphate buffer (pH 6.8) and centrifuged at 12,000×*g* and 4 °C for 20 min. The supernatant was collected for enzyme extraction and determination. The nitrogen blue tetrazole (NBT) photoreduction method was used to determine SOD activity [30]. The enzyme dosage preventing 50% NBT photochemical reduction was treated as 1 U g^− 1^. POD activity was estimated by the guaiacol method [31]. CAT activity was evaluated by the ultraviolet absorption method and defined as 1 U enzyme activity when the optical density (OD) at 240 nm min^− 1^ (A240) was reduced by 0.1 (U g^− 1^). PAL activity was determined by a previously reported method [32]. The fruit samples were extracted in 50 mM Tris buffer (pH 8.5) and centrifuged at 6000×*g* and 4 °C for 10 min to collect the supernatant. *L*-phenylalanine and 100 μL of 2 N HCl were added to the supernatant to initiate the reaction. Absorbance was recorded at 290 nm and indicated cinnamic acid formation.

### Liquid chromatograph-mass spectrometer (LC-MS) for phytohormone analysis

To determine the IAA, GA_3_, and ABA content, 3 g kiwifruit tissue was weighed, frozen, powdered in liquid nitrogen, and later analyzed by LC-MS as previously described [33]. Each treatment group was represented by three independent biological replicates.

### Statistical analysis

Statistical analyses were performed with the software package, SPSS 18.0. The student T test was used to compare defensive enzyme, phytohormone content or relative gene expression in the control and various infection groups. Statistical significance was defined as *p* < 0.05.

## Supplementary Information

**Additional file 1.**

**Additional file 2.**

## Data Availability

The kiwifruit genome database can be found in http://kiwifruitgenome.org/. The plant materials are available from the corresponding author on request.

## Abbreviations

ABA: Abscisic acid
ARF: Auxin response factor
CAT: Catalase
CTLH: C-terminal to the LisH
dpi: Days post-inoculation
EAR: Ethylene-responsive element binding factor-associated amphiphilic repression
GA_3_: Gibberellic acid
IAA: Indole-3-acetic acid
LC-MS: Liquid chromatography-mass spectrometry
LisH: Lissencephaly homologous
NBT: Nitro blue tetrazole
OD: Optical density
PAL: Phenylalanine ammonia-lyase
PDA: Potato dextrose agar
POD: Peroxidase
qRT-PCR: Quantitative reverse-transcription polymerase chain reaction
ROS: Reactive oxygen species
SA: Salicylic acid
SOD: Superoxide dismutase
TIR: Transport inhibitor response
TPD: TOPLESS domain
TPL: TOPLESS
TPR: TOPLESS-RELATED
VIGS: Virus-induced gene silencing
WUS: WUSCHEL
