# Supplementary figures and images for: Investigation of the role of AcTPR2 in kiwifruit and its response to Botrytis cinerea infection
Source: BMC Plant Biol. 2020 Dec 10;20:557. doi: 10.1186/s12870-020-02773-x (PMC7731759; doi:10.1186/s12870-020-02773-x)

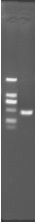

Supplement: Supplementary file 1 — Additional file 1. [file 12870_2020_2773_MOESM1_ESM.jpg]

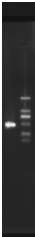

Supplement: Supplementary file 2 — Additional file 2. [file 12870_2020_2773_MOESM2_ESM.jpg]
